# Transient and Microscale Deformations and Strains Measured under Exogenous Loading by Noninvasive Magnetic Resonance

**DOI:** 10.1371/journal.pone.0033463

**Published:** 2012-03-20

**Authors:** Deva D. Chan, Corey P. Neu

**Affiliations:** Weldon School of Biomedical Engineering, Purdue University, West Lafayette, Indiana, United States of America; University of Notre Dame, United States of America

## Abstract

Characterization of spatiotemporal deformation dynamics and material properties requires non-destructive methods to visualize mechanics of materials and biological tissues. Displacement-encoded magnetic resonance imaging (MRI) has emerged as a noninvasive and non-destructive technique used to quantify deformation and strains. However, the techniques are not yet applicable to a broad range of materials and load-bearing tissues. In this paper, we visualize transient and internal material deformation through the novel synchrony of external mechanical loading with rapid displacement-encoded MRI. We achieved deformation measurements in silicone gel materials with a spatial resolution of 100 µm and a temporal resolution (of 2.25 ms), set by the repetition time (TR) of the rapid MRI acquisition. Displacement and strain precisions after smoothing were 11 µm and 0.1%, respectively, approaching cellular length scales. Short (1/2 TR) echo times enabled visualization of *in situ* deformation in a human tibiofemoral joint, inclusive of multiple variable *T_2_* biomaterials. Moreover, the MRI acquisitions achieved a fivefold improvement in imaging time over previous technology, setting the stage for mechanical imaging *in vivo*. Our results provide a general approach for noninvasive and non-destructive measurement, at high spatial and temporal resolution, of the dynamic mechanical response of a broad range of load-bearing materials and biological tissues.

## Introduction

Knowledge of the mechanical behavior of tissues is important to characterizing normal behavior in the physiological mechanical environment and identifying the design criteria for tissue engineered replacements. For diseased or degenerated tissues, the mechanical behavior could be an important indication of the damage state and a key measure for recovery and healing. For engineering biomaterials in general, the internal mechanical behavior of the material under varied loading conditions is important to characterize the material and match it to the appropriate applications.

Noninvasive and non-destructive imaging allows for complete three-dimensional mechanical characterization of load-bearing biomaterials. Bulk mechanical testing, which permits the measurement of nominal displacements and strains, cannot directly measure inhomogeneous or position-dependent material properties. Video-based techniques such as digital image correlation and speckle tracking can measure displacements and strains through the thickness of a material [1] but often require invasive cutting of the material to reveal a plane of interest. This destructive treatment alters the loading environment and likely biases the very mechanical behavior to be measured. Magnetic resonance imaging (MRI) can image the complete sample volume to provide three-dimensional data noninvasively and mechanical characterization of materials [2], [3], [4]. Previous displacement encoded MRI studies examined the motion of the myocardium [2] and the displacement of the blood vessels [5], [6] during the cardiac cycle, taking advantage of the endogenous mechanical cycling of the tissue. With displacement-encoded MRI, the displacement of a pixel is proportional to the difference in the phase component of the signal, as compared to a reference scan. This relationship between the displacement, Δx, and the difference in phase, Δϕ, is described by the following equation:
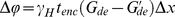
(1)where 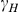 is the gyromagnetic ratio of ^1^H, *t_enc_* is the effective gradient encoding duration, 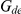 is the gradient magnitude for displacement encoding, and 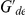 is the gradient magnitude for reference data, which was used to eliminate phase contributions common to all images [2], [7]. Displacement encoding is performed in each orthogonal direction for which displacement data is desired, using a total displacement encoding gradient moment of 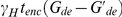.

**Figure 1:**
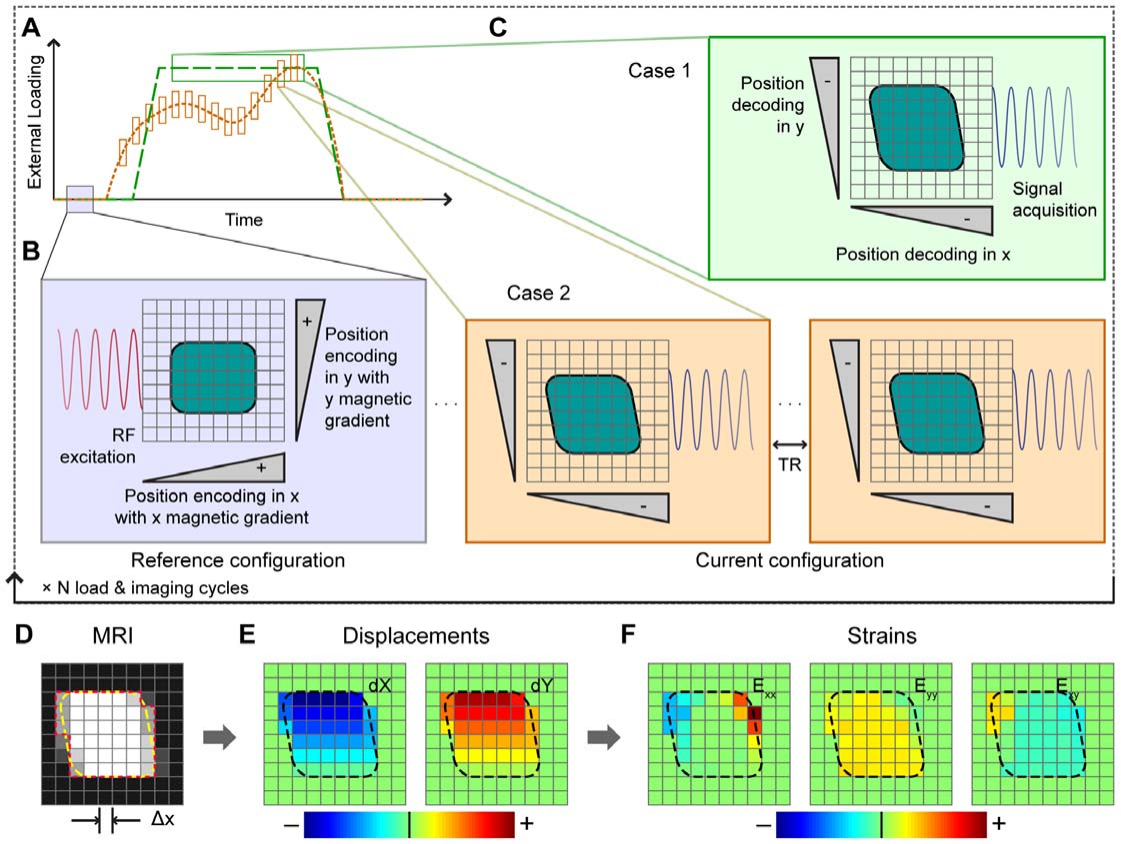
Synchronized loading and imaging for the calculation of displacements and strains using displacement-encoded MRI. Various cyclic loading regimes, including a plateau of constant load (Case 1 – long dashed green line) and a time-transient loading scheme (Case 2 – short dashed orange line) are synchronized with displacement-encoded MRI actions (*A*). Within each loading and imaging cycle, the reference configuration is encoded using radiofrequency (RF) excitation and a linear magnetic gradient (*B*). In the current deformed configuration, the new position of the material is decoded by applying another linear magnetic gradient, and signal encoded with the change in position is acquired (*C*). For Case 1, position decoding and acquisition occurs throughout the load plateau. For Case 2, decoding and partial acquisitions occur during shorter time periods within the loading scheme to produce time-resolved frames of MRI data. This acquisition scheme requires many more loading cycles but produces MRI data at frame rates limited only by TR. The spatial resolution, Δ*x* (*D*) determines the volume over which displacements (*E*) and strains (*F*) are computed for each pixel.

Because nearly all materials must be externally loaded to produce deformations (unlike, for example, myocardium), any characterization of a material's mechanical behavior during deformation using MRI requires that imaging be synchronized with the externally applied loading [7], [8] (Fig. 1). Previous studies by our group have determined displacements and strains during cyclic loading using displacement encoding with stimulated echoes (DENSE) with a fast spin echo (FSE) acquisition [7]. However, DENSE-FSE requires long acquisition times for adequate signal-to-noise ratio (SNR) of the displacement-encoded signal. DENSE with a true fast imaging with steady-state precession (TrueFISP) acquisition (combined as DENSE-FISP) allows for a higher SNR and also flexible options for imaging parameters, permitting the adjustment of both spatial and temporal resolution to the desired material and loading conditions (Fig. 1, Fig. S1).

Herein, we show that an optimized cyclic loading and DENSE-FISP imaging protocol allows for the determination of displacements in a silicone gel phantom with overall in-plane spatial resolution of 100 µm and to sub-pixel precisions (i.e. as fine as 11 µm in displacement and 0.1% in strain). The ability to segment the DENSE-FISP acquisition allows for the visualization of deformations with a temporal resolution limited only by the repetition time (TR) of the MRI sequence (Fig. S1). Additionally, we demonstrate DENSE-FISP in the various soft tissues of a cadaveric human tibiofemoral joint. Taken together, this study presents a noninvasive technique that can be used to evaluate the mechanical behavior of any cyclically loaded biomaterial at high spatial and temporal resolutions.

## Materials and Methods

### Displacement-Encoded MRI Synchronized with Exogenous Loading

Displacement-encoded imaging was implemented using a phase-cycled [9] DENSE preparation [2] and TrueFISP acquisition (Figs. 1 and S1). A displacement-encoding gradient was applied between two 90° flip radiofrequency (RF) pulses, the second of which was phase-cycled to reduce artifacts characteristic of stimulated echo preparations [9]. Application of the displacement-encoding gradient sensitized the phase component of the magnetization to its position in the reference configuration [2]. The end of the DENSE preparation and the start of the TrueFISP acquisition were separated by a variable mixing time (TM), during which the position-encoded stimulated echo was stored in the longitudinal direction. During the TrueFISP acquisition, the stored stimulated echo was brought back into the transverse plane, and a displacement-decoding gradient of the same moment was applied to decode the current configuration of the magnetization. The TrueFISP acquisition used alternating RF pulses of flip angle α = ±25° but was initiated with a α/2 pulse to accelerate the approach to a steady state magnetization. A balanced displacement-encoding gradient during the TrueFISP readout allowed data to be acquired without disrupting steady state.

**Figure 2:**
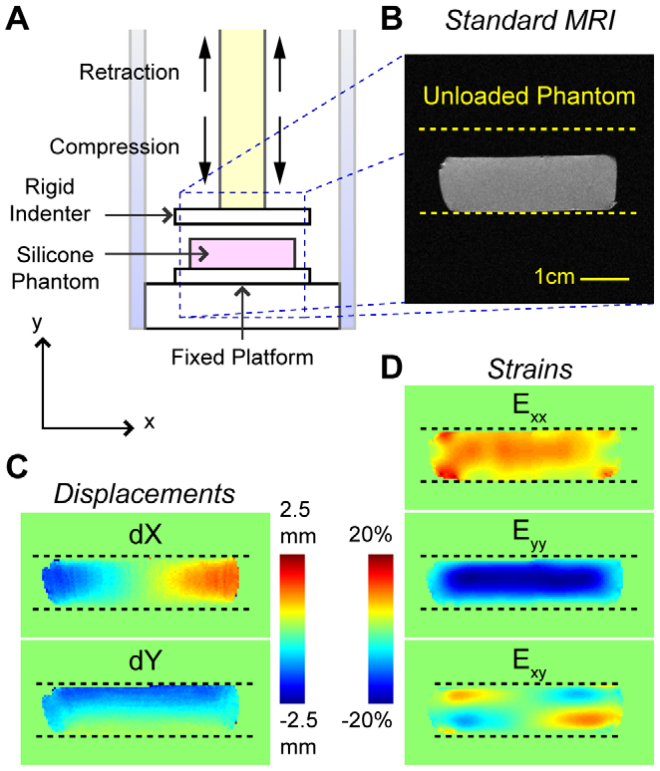
Displacements and strains determined by noninvasive DENSE-FISP. An MRI-compatible loading apparatus cyclically loads a silicone gel phantom within a 9.4T MRI system by compressing the phantom against a fixed platform and then retracting after the load plateau (*A*). The unloaded phantom was imaged using standard MRI (*B*) prior to displacement-encoded MRI. In the representative images, displacements (*C*) and strains computed from smoothed displacements, after 25 smoothing cycles, (*D*) were found to be heterogeneous.

DENSE-FISP imaging and extrinsic loading were synchronized using a digital trigger for displacement encoded MRI in a horizontal 9.4 T Biospec system (Bruker Medical GMBH, Ettlington, Germany) with a 30-cm gradient coil. A silicone phantom (Sylgard 527 dielectric gel; Dow Corning, Midland, MI) was cyclically compressed and synchronously imaged using DENSE-FISP (Fig. 2*A*–*B*). The displacement-encoding gradient was applied prior to mechanical loading and displacement decoding occurred while the material of interest was loaded. For single frame image scans (Figs. 1, Case 1, and 2), loading ramped during the mixing time of the sequence; decoding and image acquisition then occurred during the loading plateau. For cine image scans (Figs. 1, Case 2, and 3*A*), displacements that had occurred between the initial encoding gradient and the image acquisition were recorded in each movie frame. Although DENSE-FISP can be applied in arbitrary directions and for all material geometry and symmetries, for the validation studies we focused on displacements and strains in the plane of imaging. Within each displacement-encoded scan set, displacement-encoding gradients were applied in the loading (i.e. Cartesian *y*) and transverse (i.e. *x*) directions to provide in-plane displacement data. No displacement-encoding gradient was applied during reference scans, allowing for displacement-independent phase contributions to be eliminated (Eq. 1).

### DENSE-FISP Image Processing

DENSE-FISP data were processed using software (MATLAB, MathWorks, Natick, MA), as previously described [7] (Figs. 1). Briefly, phase-cycled scan repetitions were recombined using cosine and sine to eliminate (CANSEL) artifacts [9], leaving only the displacement-encoded stimulated echo signal. Phase from reference and displacement-encoded scans was zeroth and first order corrected and then unwrapped. Phase differences (Δϕ) between the reference scan and displacement-encoded scans were converted into displacements (Δ*x*) in the encoded directions (Eq. 1). Displacements were smoothed using a two-dimensional moving average filter, and Green-Lagrange strains were then computed from the smoothed displacement fields using a maximum likelihood estimate of the deformation gradient [10]. Green-Lagrange strains were chosen because they better represent the finite strains that result from large deformations, rather than Cauchy (i.e. infinitesimal) strains that are derived from small displacements.

For each repeated set of scans, SNR was computed for each imaging set by dividing the mean signal magnitude, which was computed in the full region of interest (ROI) defined by the phantom, by the mean noise signal, which was computed in the image region at least five pixels outside of the ROI. Precisions of displacement and strain were then determined as previously described [7], [11]. Within each image in a repeated set of image parameters, values for displacement and strain were obtained for nine standardized locations within the imaging phantom. Precision was then defined as the pooled standard deviation of the repeated samples over the nine different points. Displacement and strain precision was computed for each imaging parameter set and each of the smoothing cycles [12]. Within each repeated set, imaging parameters were varied depending on the experiment, as described below.

### Experimental Imaging Parameters

**Figure 3:**
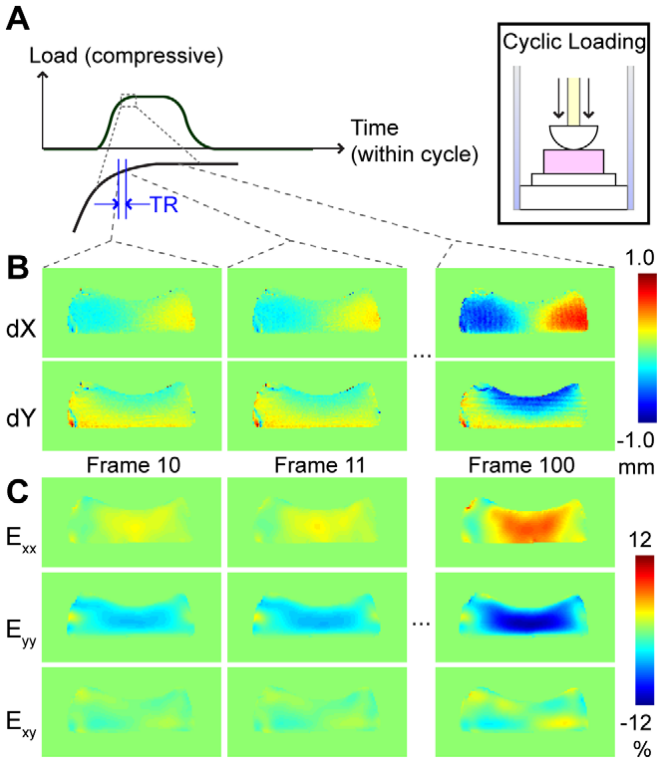
DENSE-FISP of transient mechanical behavior. High temporal resolution displacement-encoded MRI nondestructively measured displacements and strains at a sampling rate of 444 Hz (TR = 2.25 ms). Cyclic compression was applied to a silicone gel using a rounded indenter, and a 100-frame cine DENSE-FISP image was acquired during a transient portion of the loading curve (*A*). In-plane displacements (*B*) and strains (*C*) shown for frame numbers 10, 11, and 100 demonstrate that DENSE-FISP is capable of measuring transient mechanical behaviors at high temporal resolution (*B*).

The flexibility of DENSE-FISP allows for the ability to perform high temporal resolution imaging (Figs. 1 and S1). To test the limits of temporal resolution with this technique, we measured displacements and strains with a highly segmented cine acquisition (Fig. 3). Although total imaging time increased, the acquisition was segmented so that only one line of data is acquired and added to each frame with each DENSE preparation. This allowed us to fit sequential cine frames into a single loading cycle, producing a multi-frame cine acquisition that has a temporal resolution limited only by the TR. To evaluate DENSE-FISP under the conditions of higher temporal resolution, the following imaging parameters were used for a 400 µm spatial resolution, 100-frame cine displacement-encoded image: TE(echo time)/TR = 1.12/2.25 ms, field of view (FOV) = 51.2×51.2 mm^2^, slice thickness = 3.13 mm, 128 interleaved segments, TM = 150 ms, and gradient area moment = 0.65Π/mm. A cylindrical indenter was used for cyclic loading of the imaging phantom (Fig. 3*A*).

We studied the ability of the critical imaging parameter, spatial resolution, to distinguish differences in location-dependent mechanical behavior at high measurement precision. To examine the effect of resolution on the SNR and precision, the acquisition matrix size was varied while maintaining the same FOV of 51.2×51.2 mm^2^ and TM of 600 ms for repeated tests at each spatial resolution value. Imaging parameters for ten repeated 400 µm scans were TE/TR = 1.66/3.33 ms and slice thickness = 1.76 mm. Imaging parameters for ten repeated 200 µm scans were TE/TR = 1.66/3.33 ms, slice thickness = 1.76 mm, and number of averages (NA) = 1 or 4. Because imaging at a higher resolution requires a longer acquisition time, the displacement-encoded signal may decay within a single load-imaging cycle before a full acquisition can be completed. Because this was the case for resolutions finer than 200 µm, the highest resolution (i.e. 100 µm) acquisitions were segmented so that strong signal could be acquired during a shorter acquisition time but over multiple load-imaging cycles. Five repeated sets of 100 µm scans were acquired with TE/TR = 2.84/5.68 ms, slice thickness = 3.84 mm, 4 interleaved segments, and NA = 8.

We further studied the ability of another imaging parameter – the strength of the displacement-encoding gradient moment (which is proportional to encoding gradient strength×encoding time) – to directly affect the phase differences and therefore the measured displacement (Eq. 1). For this separate set of experiments, displacement encoding gradient areas moments of 0.32 Π/mm and 0.65 Π/mm were compared for a fixed 200-µm spatial resolution. Imaging parameters for these repeated scans were TE/TR = 1.66/3.33 ms, TM = 600 ms, FOV = 51.2×51.2 mm^2^, slice thickness = 3.52 mm, and NA = 1, 4, or 12.

In addition to modifying imaging parameters to improve SNR and precision, combining complementary acquisitions of DENSE-FISP images can be used to reduce the effect of unwanted image artifacts (banding). Standard TrueFISP imaging occurs with a phase advance of 180°, which results in the alternating ±α RF pulses [13]. Heterogeneous magnetic fields create characteristic banding artifacts with TrueFISP acquisitions, but choosing a different phase advance angle can shift the position of these banding artifacts. Complementary TrueFISP imaging relies on the choice of multiple phase advance angles, resulting in an average image that is affected less by banding. For the study of complementary DENSE-FISP imaging, three phase advance angles of 60°, 180°, and 300° were chosen so that banding artifacts were evenly distributed between the three sets of displacement-encoded images. Imaging parameters for complementary scans were TE/TR = 1.65/3.50 ms, TM = 600 ms, FOV = 51.2×51.2 mm^2^, slice thickness = 3.52 mm, displacement encoding gradient area moment = 0.65Π/mm, and NA = 4. During processing of these image sets, the mean complex signal at each pixel was used to reassemble an image with minimal banding artifacts. These data allowed for a comparison between basic (i.e., 180° phase advance) DENSE-FISP, two levels of signal-averaged DENSE-FISP, and signal-averaged, complementary DENSE-FISP.

To calculate strain, displacements fields must be second-order continuous, requiring the smoothing of raw displacement data. Therefore, we examined the effect of smoothing on the precision of displacement and strain values. Displacements for selected data sets (i.e. sets wherein spatial resolution, gradient moment, or complementary imaging were varied) were smoothed with a moving average filter over 0, 5, 10, 25, 50, and 100 smoothing cycles to permit the calculation of displacement and strain precisions at each of these levels.

### Displacement-Encoded MRI of a Cadaveric Tibiofemoral Joint

For the demonstration of DENSE-FISP in a human tibiofemoral joint, a fresh-frozen cadaveric knee specimen from an 83 year old female (32 kg) was prepared for imaging. The joint was obtained in compliance with the Anatomical Education Program in the Department of Anatomy and Cell Biology at the Indiana University School of Medicine. Because the tissue was cadaveric, no additional IRB approval was obtained. Tissue was excised from the knee, being careful to preserve the tibiofemoral joint and ligaments that span that joint, to allow the joint to fit into a custom MRI-compatible loading device. The tibia and femur were potted in fiberglass resin to allow for a secure attachment to the loading device, which was positioned inside the MRI scanner. The tibiofemoral joint was cyclically loaded to 220 N (70% body weight) for 1.5 seconds every 5 seconds, with loads transferred through the tibia in the inferior-to-superior direction. Displacement of the tissues in the joint was monitored by standard TrueFISP acquisitions before and during the load plateau approximately every 100 cycles. A steady-state load-deformation response was achieved in the joint [14], and tissue displacements during the load plateau of repeated scans were unchanged. Once a steady state was achieved in response to loading, MRI acquisitions were synchronized with cyclic compression of the joint.

DENSE-FISP was implemented using an encoding gradient moment of 0.65Π/mm in a coronal imaging slice for determination of in-plane displacements of a number of ROIs. DENSE-FISP acquisition parameters for the 200-µm resolution acquisition were as follows: TE/TR = 2.53/5.07 ms, TM = 800 ms, FOV = 102.4×102.4 mm^2^, slice thickness = 1.69 mm, 8 interleaved segments, and NA = 2 per scan set. Four complementary scan sets were acquired, with phase advance angles of 0°, 90°, 180°, and 270°. A complementary averaging scheme was needed because the heterogeneity in the magnetic field resulted in banding artifacts across tissues of interest. In addition, the decay of transverse magnetization (*T_2_*) has been shown in previous studies to be correlated to collagen content and architecture [15]. Therefore, *T_2_* mapping was accomplished during the load plateau using fast spin echo at effective TEs of 16, 47, 78, 110, and 141 ms. Native software (Paravision 5.1, Bruker Medical GMBH) was used to calculate the *T_2_* values on a pixel-by-pixel basis in the same ROIs.

After MRI, the tibiofemoral joint was frozen for storage and later thawed for characterization of gross anatomy and histology. Full thickness cartilage plugs were then fixed in Bouin's solution for 24 hours at 4°C, defatted using 70% ethanol for 3 days at 4°C, and then decalcified in agitated 10% formic acid in citrate for 3 days at 4°C. Specimens were then rinsed and stored in 70% ethanol prior to embedding in paraffin, sectioning, and staining with Safranin-O.

## Results

Load-synchronized DENSE-FISP permitted the measurement of heterogeneous in-plane displacements and strains in the silicone gel phantom during the load plateau (Fig. 2). DENSE-FISP at high temporal resolution (2.25 ms) resulted in the acquisition of displacements and strains as loading was increased to a plateau (Fig. 3*A*). Displacements and strains change as time within the cycle progresses, as evidenced by the similar displacement and strain fields between two adjacent frames but increased displacements (Fig. 3*B*) and strains as loading continued at a later time points (Fig. 3*C*).

**Figure 4:**
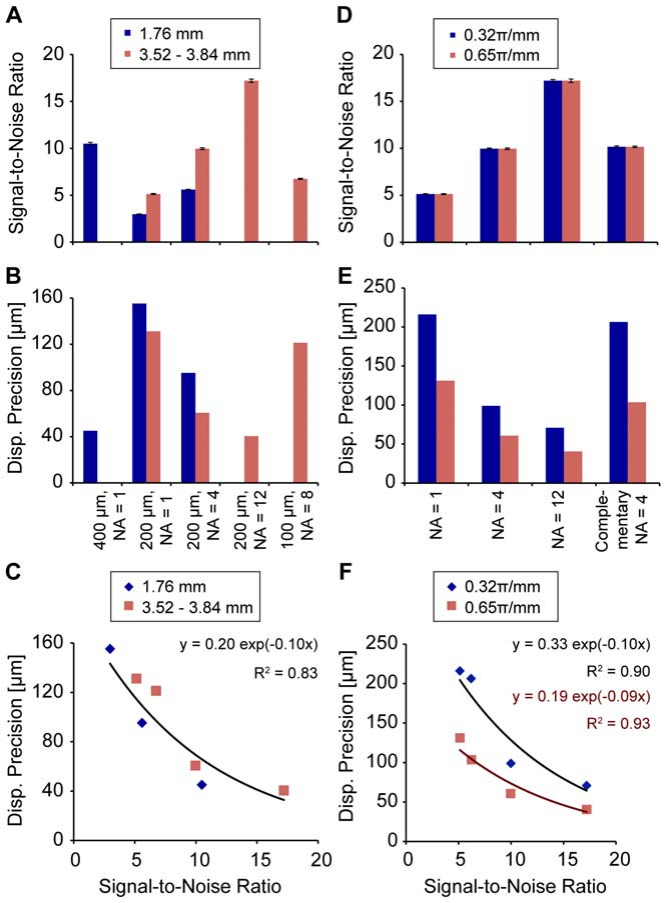
Effects of spatial resolution, slice thickness, gradient moment, and averaging. Displacement precision depends strongly on SNR of the displacement-encoded image. Finer in-plane spatial resolutions required increased NA or slice thickness (from 1.76 mm (dark blue) to 3.52–3.84 mm (light red)) to improve mean (± standard deviation) SNR (*A*) and precision (*B*). Increasing the gradient moment from 0.23π/mm (dark blue) to 0.65π/mm (light red) has no effect on SNR (*D*) but improves precision (*E*). Complementary NA = 4 acquisitions (phase advance = 60°, 180°, 300°) did not significantly affect SNR and do not show improvements to precision compared to NA = 4 scans (phase advance = 180°). SNR and precision show an exponential relationship (*C*, *F*) that can inform the choice of imaging parameters for DENSE-FISP.

The SNR and precision of displacement measurement varied with the choice of spatial resolution and other imaging parameters (Fig. 4*A*–*C*). Lower in-plane resolution, increased slice thickness, and averaging resulted in higher SNR (Fig. 4*A*). Precision depended strongly on the spatial resolution (Fig. 4*B*). Importantly, the SNR and precision were closely linked in an exponential relationship (Fig. 4*C*), with higher SNR correlated to improved precisions (R^2^ = 0.83). Overall, the raw displacement precision varied between 41 and 155 µm, depending on the choice of spatial resolution and concomitant imaging parameters.

With spatial resolution constant, averaging and the strength of the encoding gradient, as measured by the gradient moment, affected SNR and displacement precision (Fig. 4*D*–*F*). At both levels of gradient moment, a fourfold increase in the number of averages approximately doubled the SNR (Fig. 4*D*). Although no differences in SNR were associated with gradient moment (*p*>0.51), the displacement precision improved with higher gradient moment (Fig. 4*E*). As an example, at 4 signal averages and 200-µm spatial resolution, increasing the gradient moment from 0.32π/mm to 0.65π/mm improved raw displacement precision from 99 to 61 µm, although the mean SNRs were 9.98 and 9.96, respectively. With respect to the exponential relationship between SNR and precision, an increase in the gradient moment led to an increase in the scaling coefficient with little effect on the exponential curve (Fig. 4*F*).

The effect of complementary imaging was observed in the comparison between three complementary imaging sets and imaging sets with simply three times the signal averaging (Fig. 4*D*–*F*). In the homogeneous imaging phantoms, complementary acquisitions did not improve SNR nor precision beyond those of the individual constituent scans. Under a gradient moment of 0.65π/mm, SNR decreased from 9.96, at 4 signal averages with no complementary scans, to 6.23, after three complementary scans each with 4 signal averages, and precision of raw displacement changed from 61 µm to 104 µm, respectively. Additionally, scans with additional averaging (i.e. 12 averages) showed higher SNR and improved precision compared to the complementary averaging scheme (i.e. 3 complementary scans with 4 averages each), despite the same total acquisition time.

**Figure 5:**
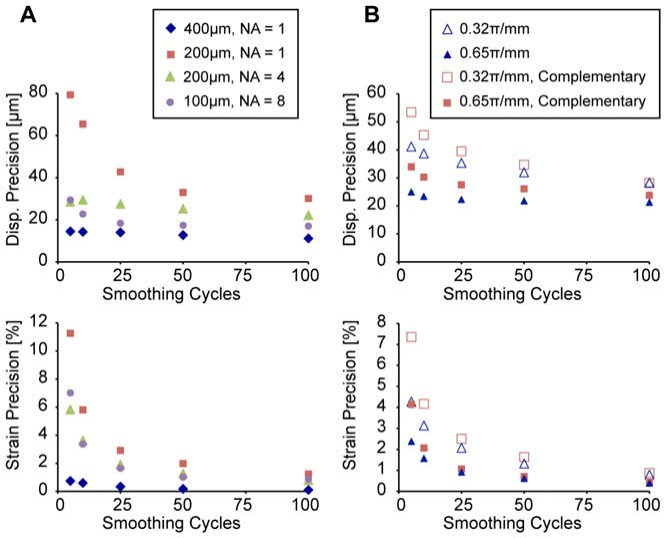
Smoothing of displacements allowed for the computer of strains. Absolute precision of both displacement (top) and strain (bottom) measurements is improved with more smoothing cycles. Across all spatial resolutions (*A*) and gradient moments (*B*), imaging parameters that show better precision in raw displacement data require fewer smoothing cycles before reaching a plateau in the precision of smoothed displacement data.

With image parameters that produce higher SNR and therefore finer precision of the raw displacement, less smoothing was required before displacement and strain precisions reached a plateau (Fig. 5). On the other hand, the displacement precision after smoothing of lower SNR scans and the strain precision of all scans did not appear to reach a plateau under the conditions tested in this study. Displacement and strain precisions after smoothing approached cellular length scales at 11 µm and 0.1%, respectively, for a 400-µm acquisition.

**Figure 6:**
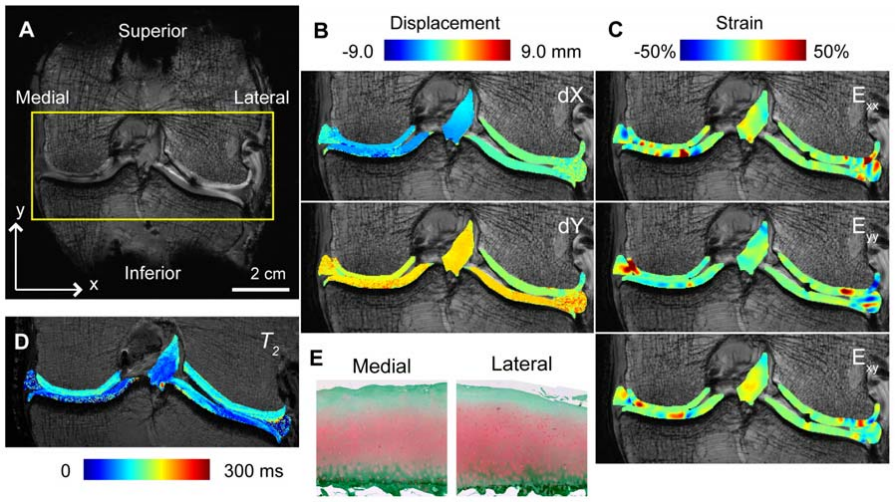
DENSE-FISP for displacements and strains in a complex system inclusive of broad *T_2_* materials. A human cadaveric tibiofemoral joint was cyclically loaded in the inferior-to-superior direction for DENSE-FISP imaging of a coronal slice (*A*). Displacements (*B*) and strains (*C*) in human tibiofemoral joint show non-homogeneous behavior in the articular cartilage, meniscus, and ligament. DENSE-FISP measures displacements in tissues that range in *T_2_* values (*D*). Cartilage sections stained with Safranin-O depict some histological signs of degeneration on the medial and lateral femoral condyles (*E*).

Finally, DENSE-FISP determined displacements and strains in a cadaveric human tibiofemoral joint (Fig. 6). The imaged coronal slice contained ROIs belonging to variable transverse relaxation time (*T_2_*) materials, including the tibial and femoral articular cartilage, the menisci, and the anterior cruiciate ligament (Fig. 6*A*). In-plane displacements of each of these soft tissue ROIs were dominated by rigid body displacements (Fig. 6*B*). Strains computed from smoothed displacements showed areas of increased strain in the medial compartment and in areas near the cartilage-meniscus interface (Fig. 6*C*). *T_2_* values were mapped in all ROIs (Fig. 5*D*) and measured as low as 67±30 ms in ligament at 9.4 T. After imaging, gross anatomy (Fig. S2) and histology (Fig. 6*E*) showed that the joint exhibited some signs of degeneration.

## Discussion

The objective of this study was to visualize transient and internal material deformation through the novel synchrony of external mechanical loading with rapid displacement-encoded MRI. We evaluated displacement-encoded MRI by DENSE-FISP for maximal temporal and spatial resolutions, using a silicone gel phantom, and demonstrated the technique in the various tissues inclusive of a cadaveric human tibiofemoral joint.

### Displacement Encoding at High Temporal Resolution

High temporal resolution cine acquisitions presented herein demonstrate that DENSE-FISP can be used to study materials with nonlinear and viscoelastic responses to applied load and to characterize the transient response of materials to any arbitrary cyclic loading scheme. To maximize temporal resolution during cyclic loading and imaging, the time between cinematic frames must be minimized. This effective sampling rate of displacement information is constrained by the RF pulse and gradient parameters and also the size of the total acquisition. Additionally, this technique would be restricted to load-deformation behaviors that remain constant across intermittent loading cycles, requiring a careful design of the loading scheme that takes into account the predicted material behavior. For example, articular cartilage would be cyclically loaded until a steady-state load-deformation response (i.e. dynamic equilibrium) is achieved, during which high temporal resolution displacement-encoded MRI could reveal the transient deformations that occur during the process of loading and unloading at frame rates in the 100 s-Hz range. Because of the inherent flexibility of the TrueFISP acquisition (Figs. 1 and S1), the number of segments into which the acquisition is split can be decreased to speed up the total time of imaging, at the cost of temporal resolution (Fig. S1).

### Imaging Parameters for Optimal Displacement and Strain Precision

With displacement-encoded MRI, displacement measurement is dependent on the phase contrast between reference and encoded scans. On the other hand, the size of the pixel, determined by the spatial resolution, dictates the volume over which these phase values are averaged. Therefore, the displacement and strain precisions reflect the repeatability, within each pixel, of measurement of the same average value. While spatial resolution is a single imaging parameter, the precision of displacement and strain measurements is affected by a number of imaging parameters, including resolution, in addition to image processing.

Minimizing the size of the pixel, or maximizing spatial resolution, is important to displacement-encoded MRI for a number of reasons. Because discrete imaging data is acquired from materials of varying geometry, partial volume effects may affect the displacement measured at pixels that include multiple materials. Therefore, the choice of a smaller pixel size allows for displacement and strain to be measured closer to the interface of materials without the confounding effects of partial volume bias. Additionally, increases to spatial resolution allow for the improved characterization of the internal deformation of a particular tissue. Location- or depth-dependence becomes simpler to discern with smaller pixels representing the same volume. However, as with most imaging parameters, increases to spatial resolution are not without tradeoffs. As the results of this study support, SNR decreases with increased resolution, requiring that signal be recovered through increases to the number of signal averages and the total acquisition time (Fig. 4A). Because of the relationship between SNR and precision shown in this study and consistent with previous studies [12], decreases in SNR reduce the ability of DENSE-FISP to detect displacements at high precision (Fig. 4C). The resulting tradeoff between high displacement precision and high spatial resolution is one that must be considered in light of the material and loading conditions being studied.

The choice in the displacement-encoding gradient also affects the precision of the measured displacements. The improved precision with an increase in gradient moment can be attributed to the ability to resolve finer differences in displacement across the same range of phase values (Eq. 1). Furthermore, this, at least in part, explains the difference in displacement precision between this study and the previous work done on a microimaging system with DENSE-FSE [7]. However, it should be noted that too great an increase in the gradient strength could lead to difficulties unwrapping the phase maps and diminished SNR due to intervoxel dephasing.

Contrary to expectations, the use of complementary acquisitions for DENSE-FISP did not improve SNR or precision. The use of other techniques to combine the complementary acquisitions were considered [16], [17], but the use of only the pixel with maximal signal or an average of all but the lowest signal pixels resulted in similar SNR and precision values (results not reported). In processing complementary images, choosing the mean signal also averages the noise, resulting in little to no change in SNR. Additionally, selecting and then averaging the complex data may affect the variability of the phase data across the same location in repeated experiments, leading to diminished displacement precision. Therefore, complementary averaging schemes may only be worth an increase in total imaging time when banding artifacts are severe enough to impair image processing (i.e., phase noise prevents phase maps from being unwrapped), as was the case for the tibiofemoral joint in this study.

In light of the various tradeoffs discussed above, the choice of imaging parameters for displacement-encoded MRI should optimize the precision of displacement measurements in order to achieve the best precision in strain calculations while necessitating the least amount of displacement smoothing. Although smoothing displacements are important for calculating the strain field, information about localized differences in displacements and unique heterogeneity in the mechanical behavior may be lost if smoothing is too severe. The results of this study on DENSE-FISP were expected to be similar to a previous study with a single set of DENSE-FSE data, which found that displacement and strain precisions improved with additional smoothing [11]. In this study, precision measurements were also improved with smoothing. Additionally, displacement and strain precisions reached a plateau with fewer smoothing cycles for scan sets wherein the imaging parameters provided higher SNR. As demonstrated by the relationships between SNR and precision, maximizing the SNR and optimizing the displacement encoding gradient moment are important to achieving good precision in displacement and therefore strain while minimizing loss of detail due to smoothing.

### Demonstration of DENSE-FISP in a Cadaveric Joint

DENSE-FISP is capable of measuring displacements not only in heterogenous specimens but also in biomaterials with variable and short *T_2_* values. Although high SNR can be easily obtained in imaging phantoms without biological activity, the use of biological tissues compounds the acquisition and interpretation of DENSE-FISP data. Firstly, average *T_2_* values in the ROIs of the tibiofemoral joint were varied and were as low as 67 ms, resulting in variable contrast throughout the ROIs. It should be noted, however, that the use of TrueFISP with rapid echo times enables deformation measurements in materials with shorter *T_2_* values. Additionally, in all materials, a steady-state load-deformation behavior should be achieved prior to imaging [8], especially when viscoelastic materials like articular cartilage are considered. Previous studies have shown that a quasi-steady state behavior can be achieved in intact joints [11], [14]. Because the tibiofemoral joint in this study required that all other tissues in the joint be removed, the joint capsule was compromised in order to fit the tibiofemoral joint into the small volume of the MRI system and loading apparatus (82 mm inner diameter). Standard TrueFISP acquisitions, however, were used to ensure that the position of all tissues during the loading plateau remained consistent over multiple loading cycles. Evaluation of the tibiofemoral joint after MRI showed signs of degeneration in both gross anatomy and histology (Fig. 5*E*), which was not unexpected considering the elderly donor. Nevertheless, these data show that DENSE-FISP is capable of measuring displacements not only in heterogenous specimens but also in biomaterials with variable and short *T_2_* values.

### Continued Advancement of Load-synchronized Displacement-encoded MRI

DENSE-FISP offers a substantial improvement over the DENSE-FSE technique previously used in synchrony with cyclic loading. The precisions of the raw displacement data (i.e. 41–155 µm range for 200 µm resolution scans) were improved from the raw displacement precision of previous studies using a DENSE-FSE acquisition at a comparable in-plane spatial resolution on a similar preclinical MRI system (132 µm precision for 250 µm spatial resolution scans [11]). Interestingly, the raw displacement precisions under some imaging paraemeters in this study were also better than the displacement precision after smoothing of displacement-encoded MRI using DENSE-FSE (65 µm after smoothing, [11]). However, when compared to precision at the same imaging resolution on a microimaging system of the same magnet strength (15.4 and 7.8 µm before and after smoothing, [7]), it is clear that there remains a tradeoff between the higher resolutions and gradient strengths available to microimaging systems and the versatility and volume capacity of larger MRI systems. Implementation of the DENSE-FISP on a microimaging system is therefore expected to permit even finer spatial resolution and improved precision in displacement and strain.

With synchronized loading and imaging, the total imaging time is directly proportional to the number of loading cycles required for the imaging acquisitions to complete. With DENSE-FSE, 256 synchronized loading and imaging cycles were required to measure in-plane displacements at an SNR of 3.5 in a silicone gel phantom [11]. However, with DENSE-FISP, an acquisition that produces a similar SNR of 4.4 only requires 48 cycles, resulting in at least a fivefold improvement in total acquisition time. In addition to the improvement in displacement precision, strain precisions achieved after smoothing in this study (i.e., 0.1%) are comparable to DENSE-FSE on a similar animal-sized MRI system (i.e., 0.2%, [11]). However, because smoothing also influences strain precision, both increased displacement precision, as demonstrated in this study, and the choice of an optimal smoothing method are required for further improvements to strain precision. Future studies should aim to continue to improve SNR, compare multiple techniques for smoothing DENSE-FISP displacement data, and to translate this technique of cyclically loading and synchronously imaging with DENSE-FISP to *in vivo* applications.

In conclusion, the ability to non-destructively measure the internal displacements and strains at high spatial and temporal resolutions has numerous applications for the study and design of biomaterials. Our current spatial resolution, 100 µm, is on a range appropriate for examining the mechanics of the extracellular environment, and physically smaller materials could be examined at even higher resolutions on micro-imaging systems. The current temporal resolution, 2.25 ms, depends on the choice in imaging parameters (i.e., RF power deposition, spatial resolution), and enables dynamic deformation tracking. The ability to measure *in situ* displacements and strains in the 100 s-Hz range could be invaluable in characterizing viscoelasticity, nonlinear mechanical behaviors, and short time deformation responses in complex (e.g. polymeric) materials.

## Supporting Information

Figure S1
**Parameters for displacement-encoded MRI synchronized with cyclic loading can be adjusted to match experimental questions and conditions.** DENSE-FISP is synchronized with applied cyclic loading to measure displacements and strains. A series of radiofrequency (RF) and magnetic gradient actions (only displacement-encoding gradient shown) comprise the DENSE-FISP pulse sequence (*A*). Displacement is encoded prior to loading with an applied gradient (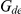) in the direction of interest and decoded during image acquisition, which begins after the mixing time (TM). In particular, the RF coil acquires a single line of data (labeled ACQ-i,j) during each repetition time (TR), for a total of *M* acquired lines to fill the data space. Because of the flexibility of TrueFISP, the acquisition can be segmented (i.e., into 4 segments) so that it is completed in less time within each cycle, at the cost of more (i.e. 4*N*) imaging cycles (*B*). To measure displacements and strains throughout a complex loading regime, only one line of data per frame is acquired each TR to maximize temporal resolution during cine acquisitions (*C*).(TIF)

Figure S2 **Standard anatomical MRI permitted registration to gross anatomy.** The slice from the standard multi-slice anatomical scan that corresponded to the physical location of the joint during the unloaded portion of the cyclic loading is shown (*A*). The tibiofemoral joint was opened for still photographs of the articular cartilage surface, menisci, and ligaments, before (*B*) and after (*C*) resection of the cruciate ligaments. Registration landmarks, including the distance between the tibial tubercles, the location of the origin of the popliteus tendon, and the width of the interconsylar notch, were identified on the standard anatomical MRI and then located within the joint, allowing for the coronal slice imaged with DENSE-FISP to be identified. The open black rectangles indicate the approximate surface areas represented in the DENSE-FISP scans. Sections of the contacting regions of the femur and tibia were then detached with a reciprocating saw before a modified coring reamer was then used to remove multiple full-thickness plugs from each of the tissue sections. The open aqua circles indicate the approximate locations from which full thickness plugs were removed for histology.
(TIF)

## References

[1] Gilchrist CL, Witvoet-Braam SW, Guilak F, Setton LA (2007). Measurement of intracellular strain on deformable substrates with texture correlation.. J Biomech.

[2] Aletras AH, Ding S, Balaban RS, Wen H (1999). DENSE: displacement encoding with stimulated echoes in cardiac functional MRI.. J Magn Reson.

[3] Muthupillai R, Lomas DJ, Rossman PJ, Greenleaf JF, Manduca A (1995). Magnetic resonance elastography by direct visualization of propagating acoustic strain waves.. Science.

[4] Osman NF, Kerwin WS, McVeigh ER, Prince JL (1999). Cardiac motion tracking using CINE harmonic phase (HARP) magnetic resonance imaging.. Magn Reson Med.

[5] Wen H, Rodriguez I, Bennett E, Vignaud A (2006). Optimization of DENSE sequence for imaging regional strain distribution in the carotid artery wall and preliminary tests in humans.. J Cardiovasc Magn Reson.

[6] Lin AP, Bennett E, Wisk LE, Gharib M, Fraser SE (2008). Circumferential strain in the wall of the common carotid artery: comparing displacement-encoded and cine MRI in volunteers.. Magn Reson Med.

[7] Neu CP, Walton JH (2008). Displacement encoding for the measurement of cartilage deformation.. Magn Reson Med.

[8] Neu CP, Hull ML (2003). Toward an MRI-based method to measure non-uniform cartilage deformation: an MRI-cyclic loading apparatus system and steady-state cyclic displacement of articular cartilage under compressive loading.. J Biomech Eng.

[9] Epstein FH, Gilson WD (2004). Displacement-encoded cardiac MRI using cosine and sine modulation to eliminate (CANSEL) artifact-generating echoes.. Magn Reson Med.

[10] Geers MGD, de Borst R, Brekelmans WAM (1996). Computing strain fields from discrete displacement fields in 2D-solids.. Int J Solids Struct.

[11] Chan DD, Neu CP, Hull ML (2009). Articular cartilage deformation determined in an intact tibiofemoral joint by displacement-encoded imaging.. Magn Reson Med.

[12] Neu CP, Hull ML, Walton JH (2005). Error optimization of a three-dimensional magnetic resonance imaging tagging-based cartilage deformation technique.. Magn Reson Med.

[13] Scheffler K, Lehnhardt S (2003). Principles and applications of balanced SSFP techniques.. Eur Radiol.

[14] Martin KJ, Neu CP, Hull ML (2009). Quasi-steady-state displacement response of whole human cadaveric knees in a MRI scanner.. J Biomech Eng.

[15] Mosher TJ, Dardzinski BJ (2004). Cartilage MRI T2 relaxation time mapping: overview and applications.. Semin Musculoskelet Radiol.

[16] Elliott AM, Bernstein MA, Ward HA, Lane J, Witte RJ (2007). Nonlinear averaging reconstruction method for phase-cycle SSFP.. Magn Reson Imaging.

[17] Bangerter NK, Hargreaves BA, Vasanawala SS, Pauly JM, Gold GE (2004). Analysis of multiple-acquisition SSFP.. Magn Reson Med.

