# Supplementary figures and images for: Transient and Microscale Deformations and Strains Measured under Exogenous Loading by Noninvasive Magnetic Resonance
Source: PLoS One. 2012 Mar 20;7(3):e33463. doi: 10.1371/journal.pone.0033463 (PMC3308970; doi:10.1371/journal.pone.0033463)

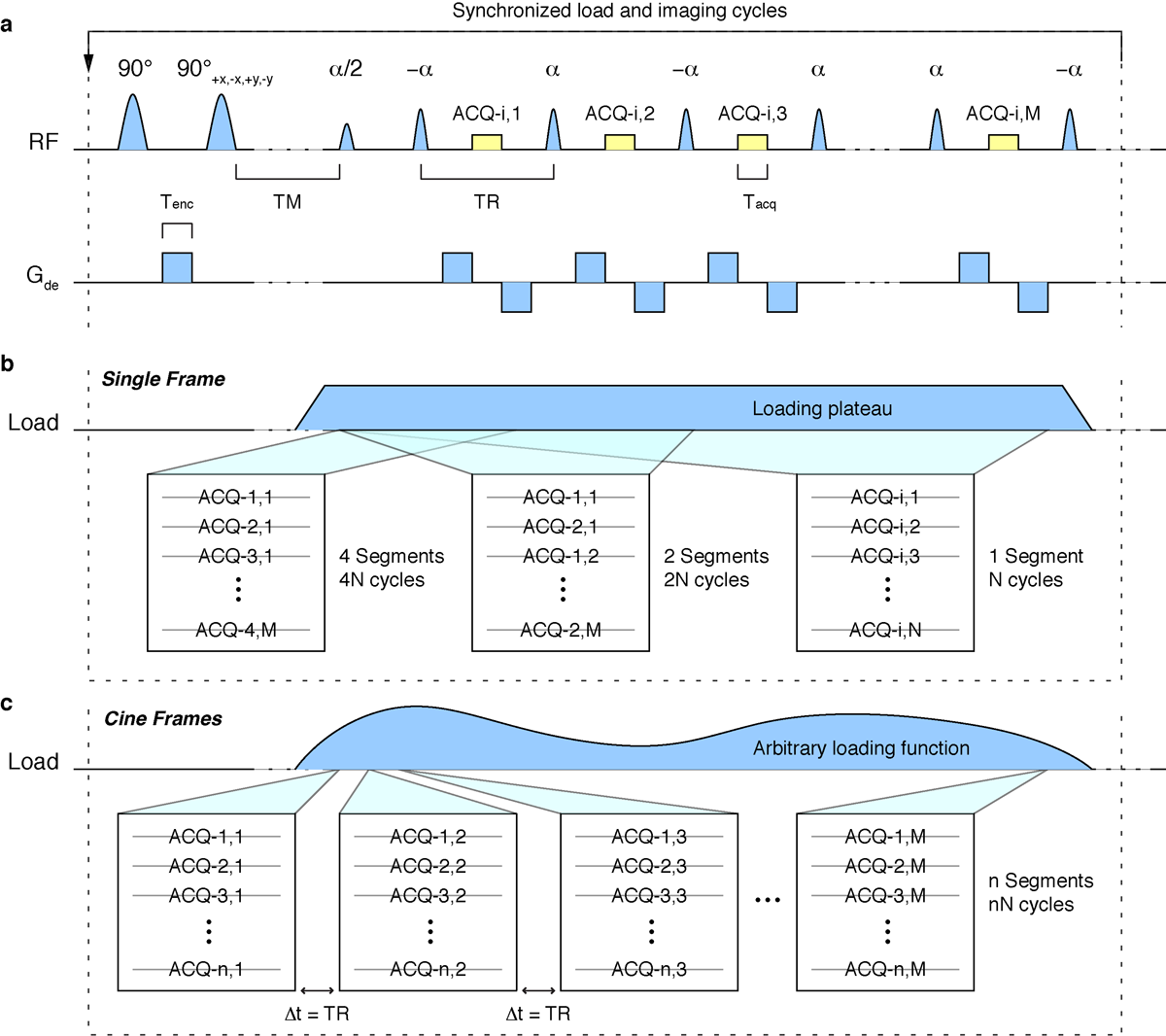

Supplement: Figure S1 — Parameters for displacement-encoded MRI synchronized with cyclic loading can be adjusted to match experimental questions and conditions. DENSE-FISP is synchronized with applied cyclic loading to measure displacements and strains. A series of radiofrequency (RF) and magnetic gradient actions (only displacement-encoding gradient shown) comprise the DENSE-FISP pulse sequence (A). Displacement is encoded prior to loading with an applied gradient () in the direction of interest and decoded during image acquisition, which begins after the mixing time (TM). In particular, the RF coil acquires a single line of data (labeled ACQ-i,j) during each repetition time (TR), for a total of M acquired lines to fill the data space. Because of the flexibility of TrueFISP, the acquisition can be segmented (i.e., into 4 segments) so that it is completed in less time within each cycle, at the cost of more (i.e. 4N) imaging cycles (B). To measure displacements and strains throughout a complex loading regime, only one line of data per frame is acquired each TR to maximize temporal resolution during cine acquisitions (C). (TIF) [file pone.0033463.s001.tif]

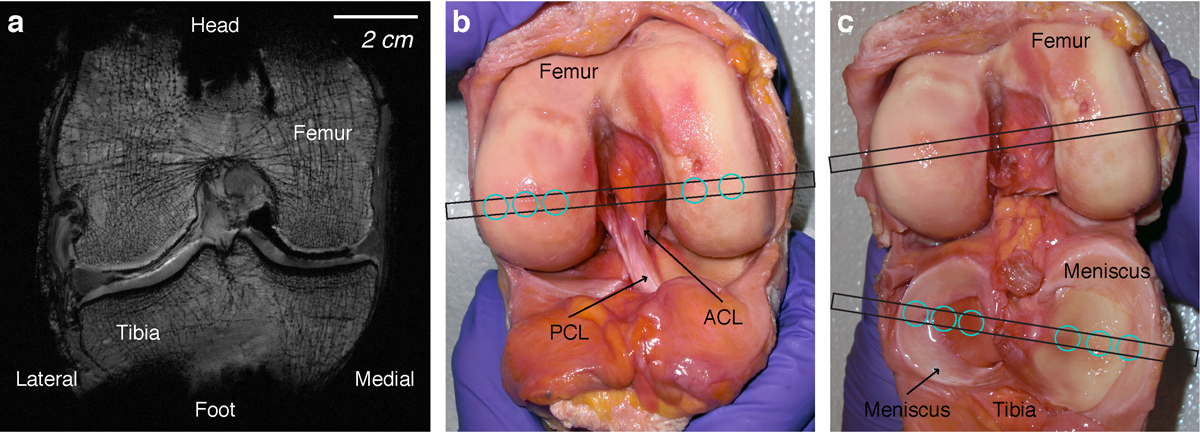

Supplement: Figure S2 — Standard anatomical MRI permitted registration to gross anatomy. The slice from the standard multi-slice anatomical scan that corresponded to the physical location of the joint during the unloaded portion of the cyclic loading is shown (A). The tibiofemoral joint was opened for still photographs of the articular cartilage surface, menisci, and ligaments, before (B) and after (C) resection of the cruciate ligaments. Registration landmarks, including the distance between the tibial tubercles, the location of the origin of the popliteus tendon, and the width of the interconsylar notch, were identified on the standard anatomical MRI and then located within the joint, allowing for the coronal slice imaged with DENSE-FISP to be identified. The open black rectangles indicate the approximate surface areas represented in the DENSE-FISP scans. Sections of the contacting regions of the femur and tibia were then detached with a reciprocating saw before a modified coring reamer was then used to remove multiple full-thickness plugs from each of the tissue sections. The open aqua circles indicate the approximate locations from which full thickness plugs were removed for histology. (TIF) [file pone.0033463.s002.tif]
